# Developing and Validating the Youth Conduct Problems Scale-Rwanda: A Mixed Methods Approach

**DOI:** 10.1371/journal.pone.0100549

**Published:** 2014-06-20

**Authors:** Lauren C. Ng, Frederick Kanyanganzi, Morris Munyanah, Christine Mushashi, Theresa S. Betancourt

**Affiliations:** 1 FXB Center for Health & Human Rights, Harvard School of Public Health, Boston, Massachusetts, United States of America; 2 Partners in Health/Inshuti Mu Buzima, Rwinkwavu, Rwanda; 3 Department of Global Health, Harvard School of Public Health, Boston, Massachusetts, United States of America; The University of New South Wales, Australia

## Abstract

This study developed and validated the Youth Conduct Problems Scale-Rwanda (YCPS-R). Qualitative free listing (*n* = 74) and key informant interviews (*n* = 47) identified local conduct problems, which were compared to existing standardized conduct problem scales and used to develop the YCPS-R. The YCPS-R was cognitive tested by 12 youth and caregiver participants, and assessed for test-retest and inter-rater reliability in a sample of 64 youth. Finally, a purposive sample of 389 youth and their caregivers were enrolled in a validity study. Validity was assessed by comparing YCPS-R scores to conduct disorder, which was diagnosed with the Mini International Neuropsychiatric Interview for Children, and functional impairment scores on the World Health Organization Disability Assessment Schedule Child Version. ROC analyses assessed the YCPS-R's ability to discriminate between youth with and without conduct disorder. Qualitative data identified a local presentation of youth conduct problems that did not match previously standardized measures. Therefore, the YCPS-R was developed solely from local conduct problems. Cognitive testing indicated that the YCPS-R was understandable and required little modification. The YCPS-R demonstrated good reliability, construct, criterion, and discriminant validity, and fair classification accuracy. The YCPS-R is a locally-derived measure of Rwandan youth conduct problems that demonstrated good psychometric properties and could be used for further research.

## Introduction

The prevalence of youth mental health disorders in sub-Saharan Africa (SSA) is estimated at approximately 10% [1]. These disorders pose a high public health burden due to their association with social and functional impairments [2] and the “youth bulge” in SSA [3]. Prevalence of conduct problems have been found to be fairly consistent across diverse cultures and contexts [4]. Among mental health disorders, conduct problems are thought to affect 3.1% to 5.7% of youth, with a median age of onset between 7 and 15 [5]. Generally, across cultures, research has found that boys have more conduct problems than girls [6], [7].

Accurate and valid tools for assessing youth conduct problems are critical, since they are associated with a host of poor outcomes including academic failure [8], violence, antisocial behavior [9], injuries, substance abuse [10], [11], [12], sexual risk behaviors [13], [14], [15], sexual and physical victimization [16], and psychiatric comorbidities, including mood and anxiety disorders [17], [18]. However, little is known about the differences in symptom presentation and perception of youth conduct problems in low and middle income countries [3]. To our knowledge, no tools for assessing conduct problems have been developed for SSA youth. This study developed and validated the Youth Conduct Problems Scale-Rwanda (YCPS-R).

The YCPS-R was developed to evaluate the efficacy of an intervention for youth with an HIV positive caregiver. Research in many countries indicates that children with HIV-positive caregivers are at increased risk for a range of mental health problems including conduct problems due to disrupted parent-child relationships, fear, and misinformation [19], [20], [21], family conflict, stigma, economic insecurity, lower education achievement, and caregiver depression and physical impairment [22], [23], [24]. Although the overall HIV prevalence rate in Rwanda has been declining and is currently estimated to be 3% [25], this trend masks the high rates of HIV among adults of caregiving age (7.9% of females between 35–39), and that one-in-six children in Rwanda is classified as vulnerable due to HIV and AIDS [26], [27]. Given the large number of children in Rwanda impacted by HIV, and their subsequent increased risk for conduct problems, developing context-appropriate measures of conduct problems, is critical for identifying children and assisting them with accessing services and care.

## Method

The YCPS-R was developed using a mixed-methods approach [28], [29] to ensure that items were culturally and contextually relevant. Qualitative studies identified and compared local symptoms and presentation profiles of youth conduct problems to scales validated in higher resource settings. Quantitative studies assessed the reliability and validity of the YCPS-R.

### Ethics Statement

Study protocols were approved by the Rwanda National Ethics Committee and the Harvard School of Public Health's Institutional Review Board. All adults gave written informed consent for themselves, all caregivers gave written informed consent for their children, and all children gave written informed assent for themselves.

### Phase 1: Identification of Mental Health Problems

To identify local expressions of conduct problems, the research team conducted two qualitative studies in Southern Kayonza district following methods previously published [30]. The first was a series of free list (FL) interviews with 31 adults (42% female) and 43 youth (47% female) selected to capture a range of gender, age, and HIV serostatus. The second gathered key informant (KI) interviews on problem themes from 47 participants. FL participants identified and described as many problems facing HIV/AIDS-affected youth in their community as possible. Problem themes representing mental health and psychosocial issues were noted. KIs, identified by FL participants and community members as being knowledgeable about psychosocial issues facing HIV/AIDS-affected youth, elaborated on problem themes and identified common cover terms that described distinct local mental health syndromes which represented specific constellations of symptoms. Ten Clinician KIs who were Rwandan mental health professionals, pediatricians, and social workers (60% women) reviewed the syndromes and associated indicators to assist with defining syndromes from a clinical perspective. One major goal of the Clinician KIs was to refine distinctions between syndromes to ensure the most accurate categorization of symptoms.

### Phase 2: Measure Selection and Development

The qualitative process described in phase one resulted in the identification of a local conduct problems syndrome and its associated symptoms. These culturally-specific symptoms were compared to items on existing measures of conduct problems to determine whether the local symptoms overlapped with indicators from measures validated in other contexts. An extensive search of youth conduct problem measures was conducted using Ovid's Health and Psychosocial Instrument database, PubMed, Google Scholar, and reference lists from identified publications. Search criteria included prior cross-cultural application with youth, strong psychometric properties, and evidence of validity in low-resource, low-literacy settings. Items from standard measures were compared for conceptual equivalence to local conduct problem indicators by U.S. and Rwandan research teams. Scales were considered a promising match if at least 50% of local indicators were captured. If a match was not made, the YCPS-R was developed solely from local indicators.

### Phase 3: Cognitive Testing

The YCPS-R was cognitive tested in Kirehe district (which borders Southern Kayonza) by six youth (aged 11–17, three females) selected from villages and schools by village leaders and principals, and six caregivers (four females). The goal of cognitive testing was to understand how participants interpreted items and chose responses. Following cognitive testing guidelines [31], bilingual (Kinyarwanda and English) Rwandan research assistants (RAs), all of whom had a bachelor's degree, assessed participant comfort with item content and asked follow-up questions to examine comprehension (“What do you think this question is asking?”), retrieval (“Tell me what you were thinking when you gave your answer?” “How did you come up with your answer?”), and judgment (“Why did you answer that way?”). RAs transcribed and translated participant answers verbatim. The study team reviewed the data and discussed items requiring revision.

### Phase 4: Reliability Study

Reliability of the YCPS-R was tested using a sample of 64 youth (51.56% female) aged 10–17 (mean age = 12.72, SD = 2.03) who were enrolled in school and living in Kirehe district. Participants were selected by village leaders and school principals. Exclusion criteria were cognitive impairment that interfered with item comprehension (as assessed by study psychologists) and refusal of caregiver consent or youth assent. The YCPS-R was administered orally by one of three Rwandan RAs and Cronbach's alpha was run to assess internal consistency. To assess whether participants would provide consistent responses to the same RA and to different RAs, one to three days later, half of the youth were administered the scale again by the same RA to assess test-retest reliability, and the remaining youth were administered the scale by a different RA to assess inter-rater reliability. Test-retest and inter-rater reliability were measured by Intraclass Correlation Coefficient (ICC) scores and Spearman's rho correlations using SPSS version 18 software [32]. Detailed demographic information has been published previously [28].

### Phase 5: Validity Study

Validity of the YCPS-R was assessed by 389 youth aged 10–17 (44.15% female; mean age = 13.41, SD = 2.24) from Southern Kayonza district and one of their caregivers. Youth were excluded if they had lived in the region for less than a month, did not speak Kinyarwanda, or had cognitive impairment as described above. In order to measure the scale's ability to differentiate between youth with and without conduct problems, the enrollment goal was to recruit at least 50 participants who likely had each syndrome and at least 50 non-cases. To identify children with and without disorders, Community Advisory Board (CAB) members and community health workers (CHWs were asked to identify youth in their villages thought to have at least one of the local mental health syndromes identified during phase one (including conduct problems), and youth thought to have no mental health disorders. In Rwanda, every family is assigned to a CHW who oversees the health and wellbeing of all of the family members. Each CHW is responsible for approximately 50 families and all CHWs were able to identify children in their villages who did or did not have at least one of the local mental health syndromes. Of the 389 youth, 53 (13.62%) were identified by either a CAB member or a CHW as having the local conduct problems syndrome, and 130 (33.42%) were reported by CAB members or CHWs to be free of any mental health syndromes.

The validity study protocol has been described previously [28]. Briefly, four bachelor's level Rwandan RAs administered the YCPS-R to youth and caregiver participants, and participants also reported (via a yes/no response) whether they thought the youth had the local conduct problems syndrome. Locally validated versions of the Center for Epidemiological Studies Depression Scale for Children (CES-DC) [28] and the World Health Organization Disability Assessment Schedule Child Version (WHODAS Child) [29] a measure of functional impairment, were also administered. One to three days later, two Rwandan bachelors-level psychologists administered the Mini International Neuropsychiatric Interview for Children and Adolescents (MINI KID) [33] including the conduct disorder (CD) and oppositional defiant disorder (ODD) subscales, to all validity study youth participants, which acted as the study's diagnostic “gold standard.” Small changes were made to the MINI KID items to make them more relevant to youth in rural Rwanda. For example, for objects that youth might use as weapons, “knives” and “guns” were retained from the original MINI KID, but “bats,” an object not typically found in Rwanda, was changed to “sticks” and “machetes.” Interviews were conducted verbally, privately and individually in participants' homes, and interviewers were blinded to the possible syndrome status of the youth. After completion of assessments, each family was given a small household gift worth approximately US$2.

Internal reliability of the YCPS-R was measured with Cronbach's alpha. Construct validity was assessed through factor analysis, with a hypothesized factor structure of one common construct. Criterion validity was assessed using correlations between the YCPS-R, MINI KID CD and ODD diagnoses, and reported presence of the local conduct syndrome, as well as between the YCPS-R and functional impairment. Discriminant validity was assessed through correlations with depression, a construct that is comorbid with conduct disorder [18], [34]. ROC curve analyses determined sensitivity and specificity trade-offs between the YCPS-R and the CD and ODD diagnoses. The Youden Index, which maximizes the difference between the true positive and false positive rate, was used to determine the optimal threshold point on the ROC curve. Differences in ROC results by sex and age were analyzed. Validity study analyses were conducted using STATA version 12 software [35].

### Phase 6: Short Form Development

To create a short form of the scale, youth responses from the validity study were analyzed for removal. Items were removed if: 1) over 90% of participants endorsed the highest or lowest response option; 2) inter-item correlations were high (the item that better represented the construct per local clinician feedback was kept, and the other was dropped); or 3) if bootstrapped factor loadings were low. The short-form was assessed for internal reliability, criterion and discriminant validity, and classification accuracy.

## Results

### Phase 1: Identification of Mental Health Problems

Analysis of the FL and KI interview data identified “uburara,” or delinquent behavior, as the local term for a cluster of youth conduct problems, including being unruly, roaming around, and engaging in sexual intercourse. Twenty-one indicators of uburara were identified (see Table 1). Some uburara indicators approximated DSM-IV-TR [36] symptoms of CD and ODD based on a face validity review by Rwandan psychologists and the U.S.-based research team. However only seven of the 21 uburara indicators corresponded to symptoms of CD or ODD, and only four of eleven CD symptoms and three of seven ODD symptoms were captured by uburara indicators. These results suggest that uburara is a related but distinct manifestation of youth conduct problems in Rwanda (see Table 1).

**Table 1:** Comparison of uburara indicators endorsed by key informants (N = 47) and DSM-IV-TR criteria for conduct disorder (CD) and oppositional defiant disorder (ODD).

Uburara Indicators (% KI endorsement)	CD Criteria	ODD Criteria
Playing dangerously/Being delinquent (74%)	---	---
Roaming around/Moving without purpose/Wandering (62%)	---	---
Being independent/Unruled (49%)	---	Actively defies or refuses to comply with adults' requests or rules
Disappearing from home (runs away)/Not wanting to stay at home (45%)	Stays out at night despite parental prohibitions, beginning before age 13/Runs away from home overnight while living in a parental home	---
Speaking rudely/insulting others (40%)	---	---
Fornicating/Engaging in Prostitution (38%)	---	---
Being undisciplined (impolite) (38%)	---	---
Stealing/Thinking of stealing (36%)	Stealing (Has stolen while confronting a victim)/Stolen items of nontrivial value without confronting the victim	---
Fighting/Being violent (34%)	Initiates physical fights	---
Taking drugs (26%)	---	---
Fearlessness (23%)	---	---
Being dirty (even if they have the means)/Doesn't bathe (23%)	---	---
Dropping out of school (even with the means to go) (21%)	Often truant from school beginning before age 13	---
Hopelessness (15%)	---	---
Having bad thoughts (15%)	---	---
Engaging in bad behaviors (13%)	---	---
Lacking a good conscience (9%)	---	---
Lacking good parenting (9%)	---	---
Not being grateful for what is given to him/her (6%)	---	---
Grumbling/Keeping a grudge (4%)	---	Angry or resentful of others/Spiteful or seeks revenge
Feeling everyone around is mocking them (2%)	---	Touchy or easily annoyed by others
---	Often bullies, threatens or intimidates others	---
---	Has used a weapon that can cause physical harm to others	---
---	Has been physically cruel to people/animals	---
---	Has forced someone into sexual activity	---
---	Has deliberately destroyed others' property/engaged in fire setting with the intention of causing serious damage	---
---	Has broken into someone else's house, building, or car	---
---	Often lies to obtain goods or favors or to avoid obligations	---
		Deliberately annoys people
---	---	Argues often
---	---	Blames others for his or her own mistakes
---	---	Often loses temper


*Note*. --- Indicates no match between uburara indicator and CD or ODD symptom.

### Measure Selection and Development

Seven standardized measures of youth conduct problems were identified from the literature search. However, none had items that overlapped at least 50% with the uburara indicators. Thus, the YCPS-R was developed solely from the uburara indicators. Fifteen indicators were selected as items for the YCPS-R per feedback from Rwandan psychologists, and “Roaming around” and “Wandering” were separated into two items, resulting in a 16-item scale. Six items were dropped because they were not seen as hallmark criteria of uburara or were thought to be largely due to comorbidity with other conditions or social context: “Disappearing from home (runs away)/Not wanting to stay at home,” “Lacking a good conscience,” “Lacking good parenting,” “Not being grateful for what is given to him/her,” “Grumbling/Keeping a grudge,” and “Feeling everyone around is mocking him/her.”

A corresponding YCPS-R caregiver report on the youth was also developed (e.g. My child engaged in bad behaviors). The YCPS-R asked youth and their caregivers to respond to each item based on how the youth felt and acted during the past week. Response options were scored on a four point Likert-type scale (0 = Not at all, 1 = A little, 2 = Some, and 3 = A lot), and the scale score was the sum (possible range 0–48). The one week time frame was selected to correspond with the time frame used in the Center for Epidemiological Studies Depression Scale for Children (CES-DC) [37], which was being validated in the same study [28]. The YCPS-R required little forward translation since local Kinyarwanda indicators were used as items. Back translation was conducted according to best-practice protocols [38], [39].

### Phase 3: Cognitive Testing

All YCPS-R items were understood by participants during cognitive testing, but six items (“I play dangerously/I am delinquent,” “I can't stay at home/I roam around,” “I use/take drugs,” “I am fearless,” “I dropped out of school although I have means/money,” “I am not clean though I have the means”) were edited to improve comprehension. For example, “I play dangerously” was removed from “I play dangerously/I am delinquent,” since participants only responded to the delinquency aspect, and “I am not clean though I have the means” was changed to specify having “hygiene materials” rather than having “means.” See Table 2 for the final YCPS-R.

**Table 2:** Long and short form versions of the YCPS-R and corresponding item numbers.

Over the last week (*Mu cyumweru gishize*):		
Long	Short	
1	1	I had bad thoughts (*Nagize ibitekerezo bibi*)
2	2	I spoke rudely (*Navugaga nabi/Nakoreshaga amagambo mabi mu mvugo*)
3	3	I was a delinquent (*Nari ikirara*)
4	4	I roamed around (*Narabungeraga*)
5	5	I was unruly/I didn't want to be ruled (*Nari icyigenge/Sinashakaga kuyoborwa*)
6	---	I wandered (*Narazereraga*)
7	---	I engaged in fornication/prostitution (*Nishoraga mu busambanyi/narigurishaga*)
8	---	I took/drank drugs (*Nanywaga ibiyobya bwenge*)
9	6	I stole (*Naribaga*)
10	7	I fought (*Nararwanaga*)
11	8	I was fearless/I didn't fear doing anything (*Nari icyihebe/Nta kintu na kimwe natinyaga gukora*)
12	9	I dropped out of school even though I didn't lack money to pay (*Navuye mu ishuri n*'*ubwo ntari mbuze amafranga yo kwishyura*)
13	---	I was not clean even though I didn't lack hygiene materials (*Nagize umwanda n*'*ubwo ntari mbuze ibikoresho by*'*isuku*)
14	---	I felt hopeless (*Nari nihebye*)
15	10	I engaged in bad behaviors (*Nishoye mu ngeso mbi*)
16	11	I was undisciplined/impolite (*Sinari mfite ikinyabupfura*)
		
		0 = Not at all (*Nta na rimwe*)
		1 = A little (*Gake*)
		2 = Some (*Rimwe na rimwe*)
		3 = A lot (*Kenshi (cyane))*

### Phase 4: Reliability Study

Results of the reliability study indicated that YCPS-R scores were skewed, with 28.13% of youth reporting no conduct problems (mean = 5.61, SD = 7.11; range = 0 to 41). Internal consistency of the reliability study data was good, with a Cronbach's alpha of .88. Of the 64 youth, 34 were enrolled in the test-retest reliability study, and the remaining 30 were enrolled in the inter-rater reliability study. The test-retest ICC was .56 and the *r*_s_ was .54, *p*<.001. The ICC of the inter-rater reliability sample was .88, and the *r*_s_ was .68, *p*<.001.

### Phase 5: Validity Study

Results of the validity study found that 23.55% of caregivers and 10.11% of youth reported that the youth had uburara, 19.95% of youth were diagnosed with CD (27.54% of males, 10.39% of females), and 3.66% were diagnosed with ODD (1.52% of males and 5.88% of females). Results from the validity study indicated that the Cronbach's alpha was .90 for the youth report and .94 for the caregiver report. YCPS-R scores were skewed, with a youth-reported mean (SD) of 8.87 (9.34), median of 6, and a range of 0 to 42.

#### Construct, Criterion, and Discriminant Validity

The YCPS-R had one common factor, accounting for 87.02% of the variance in the youth report and 88.42% in the caregiver report. YCPS-R scores were associated with CD diagnoses, and community, youth, and caregiver reported uburara diagnoses, but not with ODD diagnoses. YCPS-R scores were also highly correlated with functional impairment. The correlation between youth-reported YCPS-R and functional impairment was .58, and between caregiver reported YCPS-R was .63, indicating that the higher children scored on the YCPS-R, the poorer their functioning, and the greater their disability. YCPS-R scores had good discriminant validity, with significant correlations between conduct problems and depression ranging from .24 to .55 (see Table 3 for all correlations).

**Table 3:** Study measure correlations.

	1	2	3	4	5	6	7	8	9	10
1. Youth report YCPS-R	---									
2. Caregiver report YCPS-R	0.41***	---								
3. Youth reported uburara	0.33***	0.29***	---							
4. Caregiver reported uburara	0.27***	0.64***	0.32***	---						
5. Community referred uburara	0.12*	0.26***	0.10	0.22***	---					
6. MINI KID CD diagnosis	0.31***	0.27***	0.37***	0.26***	0.12*	---				
7. MINI KID ODD diagnosis	0.14*	0.10	0.05	0.05	-0.08	-0.05	---			
8. Youth reported depression	0.53***	0.29***	0.11*	0.14*	-0.06	0.11	0.11	---		
9. Caregiver reported depression	0.24***	0.55***	0.05	0.24***	0.09	0.11	0.02	0.38	---	
10. Youth reported functional impairment	0.58***	0.31***	0.18**	0.22***	0.10	0.11	0.08	0.52***	0.23***	---
11. Caregiver reported functional impairment	0.28***	0.63***	0.13*	0.30***	0.13*	0.16**	0.02	0.28***	0.65***	0.26***

***p<.001, **p<.01, *p<.05.

#### Sex and Age

Results of logistic regressions found that youth reported (t(364) = 4.71, *p* = .03) and caregiver reported (t(360) = 9.98, *p* = .002) YCPS-R scores were higher in males than in females. Similarly, males were more likely to be diagnosed with CD (B = −1.19, *p*<.001), to have community referred uburara (B = 1.61, *p*<.001), caregiver reported uburara (B = −.95, *p* = .001), and to have self-reported uburara (B = −.98, *p* = .02) than females. However, females were more likely to be diagnosed with ODD than males (B = 1.40, *p* = .04). Results of logistic regressions indicated that age did not predict diagnosis of CD or community, caregiver, or youth reported uburara diagnoses (*p*s>.24), and results of linear regression found that age did not predict youth or caregiver reported YCPS-R scores (*p*s>.12).

#### Classification Accuracy

**Figure 1:**
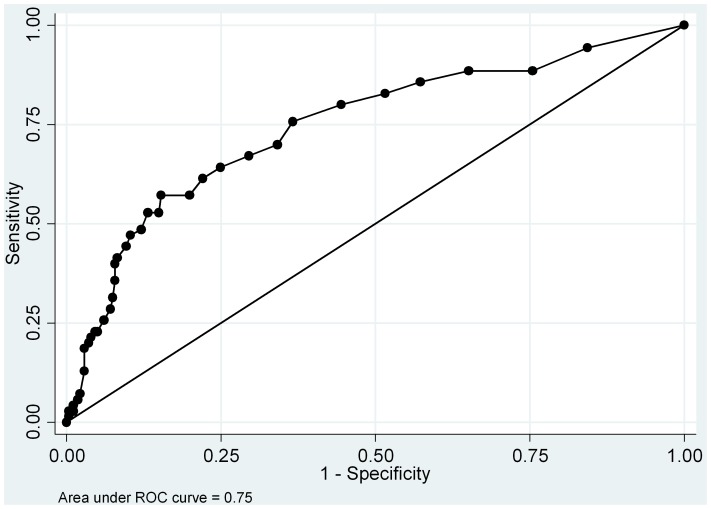
Youth reported YCPS-R scores predicting conduct disorder.

**Figure 2:**
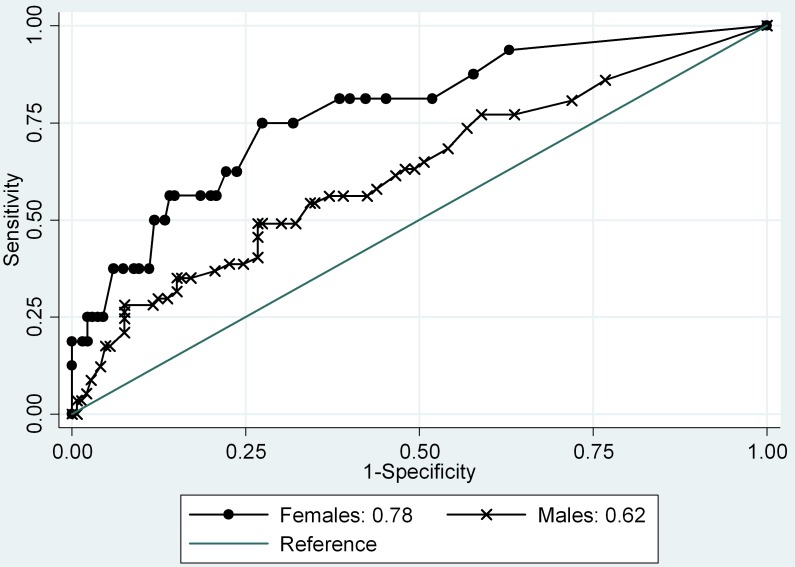
Caregiver reported YCPS-R scores predicting conduct disorder by sex.

ROC analyses assessed the ability of the YCPS-R to discriminate between youth diagnosed and not diagnosed with CD. Since only 3.66% of youth were diagnosed with ODD, ROC analyses were restricted to CD diagnoses. Since sex predicted YCPS-R and CD, ROC analyses were run by sex. Age was not included as a covariate, since age did not predict YCPS-R or CD. There were no differences in the youth report YCPS-R predicting CD under the curve (AUC) for males and females (*X* ^2^ = .61, *p* = .44). The AUC was .75 (95% CI = .68–.82), which is considered reasonably accurate [40] (see Figure 1). The Youden Index optimal threshold was 13, which provided a sensitivity of 57.14% and a specificity of 84.70%. The AUC for the caregiver report YCPS-R was higher for females (AUC = .78, 95% CI = .65–.90) than males (AUC = .62, 95% CI = .54–.71) (*X* ^2^ = 3.95, *p*<.05) (see Figure 2). The optimal threshold for the caregiver report was nine for females (sensitivity = 75.00%, specificity = 72.59%) and 16 (sensitivity = 54.39%, specificity = 65.75%) for males.

### Phase 6: Short Form of the YCPS-R

Analysis of validity study item distributions indicated that more than 90% of responses to the items “I engaged in fornication/prostitution” and “I took/drank drugs” were 0 = Not at all, and so they were dropped from the short-form scale. The items “I roamed around” and “I wandered” were conceptually similar and highly correlated (*r*_s_ = .72, *p*<.001). The local assessment team thought “I roamed around” better represented the uburara construct, and so it was kept, while “I wandered” was dropped. The items “I was not clean even though I didn't lack hygiene materials” and “I felt hopeless” loaded least well onto the construct (λ = .52 and .62, respectively), and so these items were dropped. See Table 2 for the complete short-form scale.

Cronbach's alpha for the short form was .89 for the youth report and .93 for the caregiver report. The short form also demonstrated good criterion validity, as the youth and caregiver reports were correlated with youth (*r*_s_ = .23, *r*_s_ = .23 respectively) and caregiver (*r*_s_ = .28, *r*_s_ = .62) reported functional impairment, CD diagnoses (*r*_s_ = .35, *r*_s_ = .28) and community (*r*_s_ = .18, *r*_s_ = .28), caregiver (*r*_s_ = .28, *r*_s_ = .67), and youth reported uburara diagnoses (*r*_s_ = .34, *r*_s_ = .29) (all *p*s<.005). The youth and caregiver report short form also demonstrated good discriminant validity, as they were moderately correlated with youth (*r*_s_ = .46, *r*_s_ = .28) and caregiver reported youth depression (*r*_s_ = .21, *r*_s_ = .51, all *ps*<.001).

ROC analyses found no difference between the AUC for males and females for the youth report short form (*X* ^2^ = .47, *p* = .49). Classification accuracy of the youth report short form was fair (AUC = .75, 95% CI = .69–.82), and the optimal threshold was five (sensitivity = 77.14%, specificity = 63.35%). The AUC for the caregiver reported short form was better for females (AUC = .79, 95% CI = .68–.91) than males (AUC = .63, 95% CI = .54–.71) (*X* ^2^ = 4.90, *p* = .03). The optimal threshold for the caregiver reported short form was nine for females (sensitivity = 68.75%, specificity = 80.00%) and 14 for males (sensitivity = 49.12%, specificity = 71.92%).

## Discussion

The psychometric properties of the YCPS-R were strong, with one common factor and good internal, test-retest, and inter-rater reliability. The measure also demonstrated good criterion validity, as scores were positively associated with functional impairment, and self-reported, caregiver-reported, and community-referred uburara diagnoses, as well as “gold standard” CD diagnoses from the MINI KID. The YCPS-R also demonstrated good discriminant validity by being correlated, but not collinear with, depression.

The classification accuracy of the youth report YCPS-R and the caregiver report for females was fair, but the classification accuracy of the caregiver report for males was poor. The fair to poor classification accuracy may reflect differences between uburara and the MINI KIND CD diagnosis that was used as the gold standard. Indeed, the CD diagnosis may not be appropriate in the Rwandan context, as it was only modestly correlated with the YCPS-R and uburara diagnoses, and was not correlated with youth reported functional impairment. Therefore, the YCPS-R may be best used as a continuous measure of conduct problems rather than a diagnostic tool. Youth with elevated scores on the YCPS-R would be expected to have more functional impairment, but cannot be assumed to meet criteria for CD. 

Some study limitations must be noted. The validity sample was a purposive sample, with approximately 67% of children referred for at least one mental health disorder, and the other 33% identified as not having any mental health disorder. The non-random sampling limits the generalizability of results. Additional studies are needed to examine the psychometric properties of the YCPS-R in a representative sample of Rwandan youth. While the YCPS-R was also developed for use with HIV-affected youth and their families, the uburara construct itself is expected to generalize to non-HIV affected youth. Additionally, it should be emphasized that the qualitative and quantitative results indicate that while uburara is related to CD and ODD, it is a distinct presentation of conduct problems in Rwanda, and not entirely equivalent to DSM-IV-TR CD or ODD criteria [36].

To our knowledge, this is the first measure developed in SSA for assessing youth conduct problems. The qualitative results indicated that the youth conduct problems in Rwanda are related to, but distinct from, conduct problem symptoms identified in western contexts, and that measuring them warrants the development of a new scale, rather than adaptation of an existing measure. The results lend support to the argument that the construct validity of scales must be assessed when the scales are used in new contexts, in addition to assessing the scale reliability and factor structure [41]. Moreover, this study provides an example of the importance, and process, of ensuring that measures are culturally relevant and appropriate for the settings in which they are used [42], [43].

### Data Availability Statement

Given the sensitive nature of the data and the need to protect research participant confidentiality, the data used in this study has not been deposited in a public repository. However, the de-identified minimal data used to reach the conclusions and replicate the analyses described in this manuscript is available upon written request to, and after review and approval by, the Harvard School of Public Health's Office of Human Research Administration.
